# Influence of internal climate variability on Indian Ocean Dipole properties

**DOI:** 10.1038/s41598-018-31842-3

**Published:** 2018-09-10

**Authors:** Benjamin Ng, Wenju Cai, Tim Cowan, Daohua Bi

**Affiliations:** 1 Centre for Southern Hemisphere Oceans Research (CSHOR), CSIRO Oceans and Atmosphere, Hobart, Tasmania Australia; 2 CSIRO Climate Science Centre, Aspendale, Victoria Australia; 3 School of Geosciences, The University of Edinburgh, Edinburgh, Scotland; 4 University of Southern Queensland & Bureau of Meteorology, Melbourne, Victoria Australia

## Abstract

The Indian Ocean Dipole (IOD) is the dominant mode of interannual variability over the tropical Indian Ocean (IO) and its future changes are projected to impact the climate and weather of Australia, East Africa, and Indonesia. Understanding the response of the IOD to a warmer climate has been largely limited to studies of individual coupled general circulation models or multi-model ensembles. This has provided valuable insight into the IOD’s projected response to increasing greenhouse gases but has limitations in accounting for the role of internal climate variability. Using the Community Earth System Model Large Ensemble (CESM-LE), the IOD is examined in thirty-five present-day and future simulations to determine how internal variability influences properties of the IOD and their response to a warmer climate. Despite small perturbations in the initial conditions as the only difference between ensemble members, significant relationships between the mean state of the IO and the IOD arise, leading to a spread in the projected IOD responses to increasing greenhouse gases. This is driven by the positive Bjerknes feedback, where small differences in mean thermocline depth, which are caused by internal climate variability, generate significant variations in IOD amplitude, skewness, and the climatological zonal sea surface temperature gradient.

## Introduction

Interannual variability in the Indian Ocean (IO) is dominated by the Indian Ocean Dipole (IOD) which develops in austral winter (June, July, August; JJA) and peaks in spring (September, October, November; SON)^1,2^. During the positive phase of the IOD, sea surface temperatures (SSTs) are anomalously warm in the western equatorial IO (WEIO) and cold in the eastern equatorial IO (EEIO). Opposite anomalies occur during the negative phase, with cooler than normal SSTs in the WEIO and warm SSTs in the EEIO region. These SST anomalies (SSTAs) alter the atmospheric circulation over the tropical IO, impacting several countries surrounding the basin. The consequences of an IOD event were most widely felt in 1997 when an extreme positive IOD occurred^1–3^. This event caused heavy rainfall over parts of East Africa, culminating in widespread flooding and the spread of infectious diseases^3,4^. On the eastern side of the IO, Australia and Indonesia experienced drier than normal conditions, resulting in drought and bushfires^3^. This 1997 extreme positive IOD coincided with an extreme El Niño and the widespread consequences of these extreme events led to significant research on the IOD. It is now known that these impacts over East Africa^5–8^, Australia, and Indonesia^9–13^ typically occur during positive phases of the IOD. Negative IODs impact these countries in an opposite manner (i.e., East Africa experiences drier than normal conditions whilst Australia and Indonesia are wetter), but the impacts from these events tend to be relatively weaker compared to positive IODs. This is caused by the positive skewness of the IOD where positive events tend to be stronger in amplitude than negative events^14–16^. Nevertheless, it has been suggested that an absence of negative IOD events may explain Australia’s recent droughts such as the Millennium Drought^11^.

The severe and widespread impacts of the IOD highlights the importance of understanding its response to a warmer climate and there have been several studies examining this response using either a single coupled general circulation model (CGCM) or a multi-model ensemble^17–22^. Models partaking in the Coupled Model Intercomparison Project phase 5 (CMIP5) tend to exhibit a positive IOD-like warming pattern over the tropical IO, with weaker (stronger) warming in the EEIO (WEIO) and an easterly wind trend^17,18^. This response is driven by the projected weakening of the Walker circulation in a warmer climate, which is behaviour displayed by the majority of models^23–26^. As the Walker circulation weakens, extreme positive IODs such as the 1997 event are projected to increase in frequency by almost three times by the end of the 21^st^ century^3^. The weakening of the mean westerly winds over the equatorial IO allows reversals of the wind and ocean currents to occur more often, causing strong cooling in the EEIO.

Analysis of individual CGCMs or multi-model ensembles does not account for internal climate variability as each model has its own internal variability and biases. To understand and capture internal climate variability, a single CGCM with an ensemble of simulations with the same external forcings must be used. This differs from perturbed physics ensembles where parameters are varied in a model which in turn generates different responses to the climate system. Projects involving large ensembles have been performed previously and this has allowed studies in climate extremes, trends, and uncertainty in projections^27–30^.

The influence of internal climate variability on the IOD and its properties has not been widely studied although a recent paper has examined the role of internal climate variability in the IOD’s response to a warmer climate^30^. It is suggested that the uncertainty in IOD amplitude change due to internal variability may account for up to 50% of the inter-model uncertainty in CMIP5. However, there appears to be no correlation between IOD amplitude variability and mean state change in the Community Earth System Model Large Ensemble (CESM-LE)^30^. This differs from CMIP5 where the uncertainty (or spread) of models’ IOD amplitude is associated with changes in the mean state^18,30^. Here, we examine the influence of internal climate variability on properties of the IOD through variations in the mean state of the tropical IO. We show instead that small differences in the mean thermocline depth, which arise due to internal climate variability, can influence projected changes in IOD properties.

## Results

**Figure 1:**
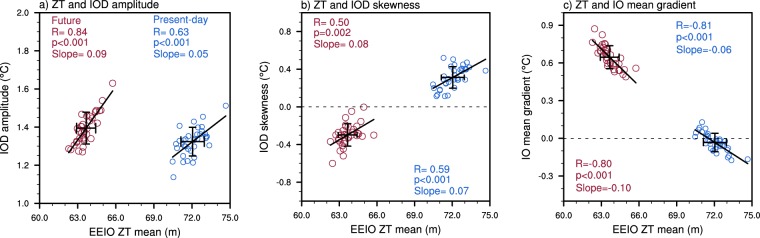
Relationship between thermocline depth and IOD properties. (**a**) Present-day (blue circles) and future (red circles) SON relationship between mean eastern equatorial Indian Ocean (EEIO) thermocline depth (ZT) and IOD amplitude. (**b**,**c**) As in (**a**) but for IOD skewness and the IO mean gradient, respectively. The diagonal black lines represent the line of best fit and the error bars indicate 1 standard deviation from the multi-member ensemble mean.

In CESM-LE, the mean EEIO thermocline depth shows a strong and significant relationship with IOD properties such as amplitude (i.e., standard deviation), skewness, and the mean zonal SST gradient (Fig. 1). This significant correlation occurs for both the present-day (blue circles) and future simulations (red circles). When the climatological thermocline is deeper, IOD amplitude tends to be stronger (Fig. 1a) and its skewness is more positive (Fig. 1b), resulting in a strong positive correlation. The mean depth of the thermocline influences IOD amplitude and skewness through the positive Bjerknes feedback which involves the SST response to the thermocline anomalies, the wind response to SSTAs, and the thermocline response to wind anomalies^31^. During a positive IOD, the thermocline in the EEIO shoals, bringing cold deep water closer to the surface which reinforces the cold SSTA and the basin-wide zonal temperature gradient. The stronger zonal temperature gradient in turn strengthens the easterly wind anomalies over the central equatorial IO (CEIO; 5°S-5°N, 70°E-90°E), deepening the thermocline in the WEIO whilst shoaling it in the EEIO. Therefore, in simulations where the mean EEIO thermocline is deep, anomalous shoaling of the thermocline is larger and reinforced through the Bjerknes feedback, resulting in a greater IOD amplitude.

The Bjerknes feedback also causes positive IOD events to be relatively larger in amplitude than negative IODs (i.e., positive IOD skewness). SSTs respond more strongly to an anomalously shoaling thermocline when the climatological thermocline is deep and hence simulations with a deeper mean thermocline tend to exhibit more positive IOD skewness (Fig. 1b). The IO mean gradient (i.e., the zonal mean SST gradient) displays a significant negative correlation with climatological thermocline depth (Fig. 1c). A climatologically deeper EEIO thermocline is associated with warmer mean SSTs in the east and therefore the west-minus-east zonal SST gradient tends to be weaker. This behaviour is associated with a stronger (i.e., more westerly) climatological zonal wind along the equator as these winds act to deepen the thermocline in the east, reducing the thermocline slope and thereby weakening the IO mean gradient.

It is clear from Fig. 1 that the mean thermocline shoals in response to a warmer climate (e.g., compare blue and red circle values on the x-axis). This is due to the projected weakening of the Walker circulation which reduces the climatological westerlies over the CEIO and allows the mean EEIO thermocline to shoal^23–26^. As such, there is a significant difference between the present-day and future IOD skewness and gradient (Fig. 1b,c; compare values on y-axis). In the future period, the shallower climatological thermocline reduces the response of cold SSTAs to an anomalously shoaling thermocline during a positive event. This weakens the amplitude of positive IODs and thus positive IOD skewness is reduced. Similarly, the shoaling of the climatological thermocline in the east leads to a strengthened (more positive) IO mean gradient as there is little change in mean thermocline depth in the west. In CESM-LE, the response of the EEIO thermocline to increasing greenhouse gases is approximately ten times greater than that over the western IO (Table S1) and faster (slower) warming over the western (eastern) IO occurs. Previous studies have used the slope (or gradient) of the thermocline across the tropical IO^32^, however in this analysis, we have used EEIO thermocline depth as the IOD is dominated by its eastern pole. Using the climatological thermocline slope does not affect the results considerably (Fig. S1). Given that the EEIO dominates the IOD, and that the response to increasing greenhouse gases is also largely over the east, we focus on this region. It is clear from Fig. 1 that despite the runs being identical in physics and parameterisation, internal climate variability is able to influence the mean state and through this, properties of the IOD.

**Figure 2:**
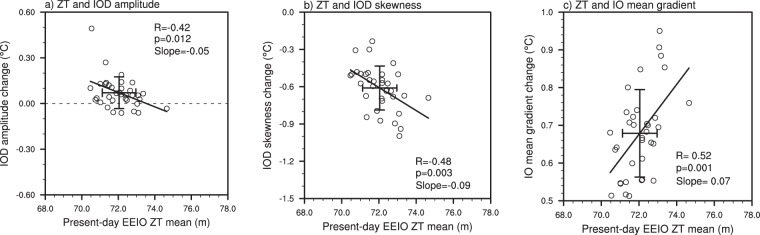
Relationship between present-day thermocline depth and the change in IOD properties. (**a**) Scatter plot showing the relationship between the present-day mean EEIO thermocline depth and the future-minus-present-day change in IOD amplitude. (**b**,**c**) As in (**a**) but for IOD skewness and IO mean gradient, respectively. The diagonal black lines represent the line of best fit and the error bars indicate 1 standard deviation from the multi-member ensemble mean.

The spread of IOD properties in the future simulations (e.g., Fig. 1, red circles) indicates that internal climate variability is able to generate variations in the IOD’s response to increasing greenhouse gases. This is important for understanding climate projections as future changes may be influenced by internal variability. To highlight the influence of the present-day mean state on projections of IOD properties, we show the relationship between the present-day climatological thermocline depth and the future-minus-present-day change in IOD properties (Fig. 2). When the present-day climatological thermocline depth is deeper, the change in IOD amplitude tends to be smaller or even negative but overall, the ensemble mean change in amplitude is close to zero (Fig. 2a). This may be caused by the different response of the WEIO and EEIO to a warmer climate with the CESM-LE simulated EEIO (WEIO) SST exhibiting an increase (decrease) in amplitude change^30^.

A deeper present-day thermocline is associated with a larger change in IOD skewness (Fig. 2b), with future skewness generally becoming negative whilst present-day skewness is positive (i.e., compare red and blue circles in Fig. 1b). This shows that changes in IOD skewness may arise due to variations in mean thermocline depth and that the deeper the mean present-day thermocline, the larger the change from positive to negative IOD skewness. Simulations with a deep present-day mean thermocline tend to project a larger change in climatological thermocline depth as a deeper thermocline has more ‘room’ to shoal (Fig. S2). This highlights the importance of the present-day mean state in influencing the response of the tropical IO to a warmer climate. As the climatological thermocline becomes shallower, it becomes more sensitive to anomalous deepening which occurs during a negative IOD event. In contrast the sensitivity to a shoaling thermocline (i.e., a positive IOD) is reduced and therefore IOD skewness becomes more negative in a warmer climate. The change in the IO mean gradient displays a positive correlation with present-day thermocline depth (Fig. 2c), meaning simulations where the mean depth is deeper tend to have a larger change in the zonal west-minus-east SST gradient. Given that runs with a deeper present-day mean thermocline tend to shoal more in the mean state, this in turn reduces the rate of warming in the EEIO and therefore, the climatological zonal temperature gradient in the future becomes larger (more positive) with the WEIO warming faster than the EEIO.

**Figure 3:**
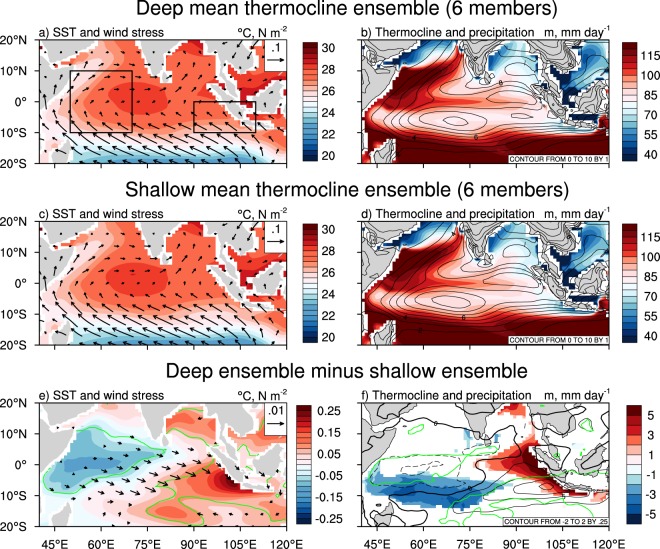
Present-day mean state over the tropical Indian Ocean for simulations with a deep or shallow thermocline. (**a**) Ensemble mean present-day climatological SSTs and wind stress from six simulations with a deep mean EEIO thermocline. (**b**) As in (**a**) but for thermocline depth and precipitation. (**c**,**d**) As in (**a**,**b**) respectively, but for six simulations with a shallow mean EEIO thermocline. (**e**) The difference in mean SSTs and wind stress between the two six-member ensembles (i.e. (**a**) minus (**c**)). (**f**) As in (**e**) but for thermocline depth and precipitation. Only vectors where either the zonal or meridional wind stress difference is significant at the 99% confidence interval are shown in (**e**). Only regions where the difference in thermocline is significant at the 99% confidence interval are shown in (**f**). The green contours in (**e**) and (**f**) represent regions where the SST and precipitation difference is significant at the 99% confidence level, respectively.

Comparing runs with a climatologically deep thermocline against simulations with a shallow thermocline can provide insight into influence of the mean state on IOD properties and this also highlights their differing response to increasing greenhouse gases. Two smaller ensembles are created to contrast these differences and the simulations in these ensembles are selected based on their deviation from the mean. Runs in the deep ensemble exhibit a present-day climatological thermocline depth greater than one standard deviation from the multi-member ensemble mean (i.e., the right-most blue circles outside of the x-axis error bars in Fig. 1a). Similarly, the shallow ensemble is comprised of runs with a present-day mean depth which is less than the ensemble mean minus one standard deviation (i.e., the left-most blue circles outside of the x-axis error bars in Fig. 1a). Analysing these two smaller ensembles shows how the positive Bjerknes feedback assists in the present-day mean state. That is, the deep thermocline ensemble exhibits warmer (colder) SSTs in the eastern (western) equatorial IO and the mean zonal wind in the CEIO is more westerly (Fig. 3a,e). The depth of the climatological thermocline along the equator extends further eastward in this ensemble, reaching the coast of Sumatra (Fig. 3b,f). Due to the warmer mean SSTs in the east, precipitation is larger over the EEIO, consistent with the wind and thermocline behaviour.

**Figure 4:**
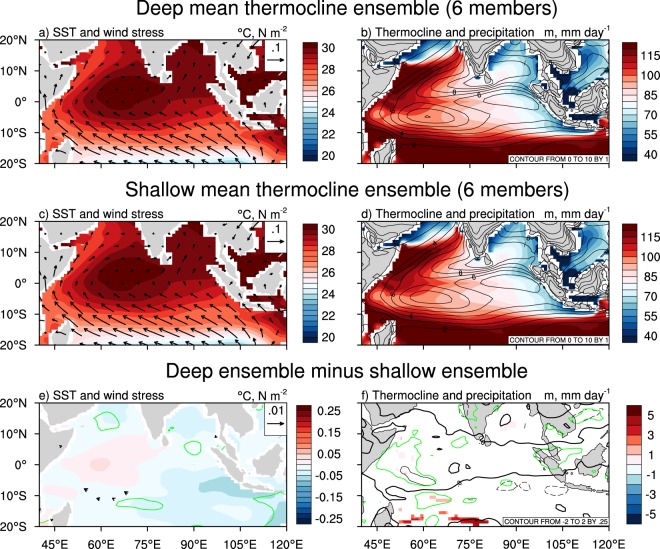
Future mean state over the tropical Indian Ocean for simulations with a present-day deep or shallow thermocline. (**a**) Ensemble mean future climatological SSTs and wind stress from six simulations with a deep present-day mean EEIO thermocline (i.e., the same simulations used in Fig. 3). (**b**) As in (**a**) but for thermocline depth and precipitation. (**c**,**d**) As in (**a**,**b**) respectively, but for six simulations with a shallow present-day mean EEIO thermocline. (**e**) The difference in mean SSTs and wind stress between the two six-member ensembles (i.e. (**a**) minus (**c**)). (**f**) As in (**e**) but for thermocline depth and precipitation. Only vectors where either the zonal or meridional wind stress difference is significant at the 90% confidence interval are shown in (**e**). Only regions where the difference in thermocline is significant at the 90% confidence interval are shown in (**f**). The green contours in (**e**) and (**f**) represent regions where the SST and precipitation difference is significant at the 90% confidence level, respectively. Fig. S3 shows the difference without significance highlighted.

This mean state with a deeper EEIO thermocline is more favourable for the growth of positive IOD events. When a positive (negative) IOD event occurs, it weakens (strengthens) the mean westerly winds which allows the climatologically warm SSTs in the east to be advected westward (eastward) whilst also shoaling (deepening) the EEIO thermocline. Therefore, a mean state similar to that of the deep ensemble allows positive IODs to grow larger in amplitude as the EEIO thermocline can shoal more when the climatological westerlies are weakened. For the shallow thermocline ensemble, the response during a positive IOD is weaker. Relatively colder mean SSTs in the EEIO and more easterly (or less westerly) mean winds over the CEIO can be observed (Fig. 3e), making it more difficult to generate a strong response during a positive IOD event. The differences between the two ensembles are clearly shown in Fig. 3e,f. Using these same runs from the present-day ensembles to compare the future climatology highlights the increased response of models with a deep present-day mean thermocline. In a warmer climate, the deep and shallow ensembles show similar mean SSTs, winds, thermocline, and rainfall (Fig. 4) as the climatological thermocline in the deep ensemble tends to shoal more in response to increasing greenhouse gases. Given this stronger response and increased similarity between the two ensembles, the deep-minus-shallow ensemble difference is insignificant over most of the tropical IO at the 90% confidence interval (Figs. 4e,f, and S3).

**Figure 5:**
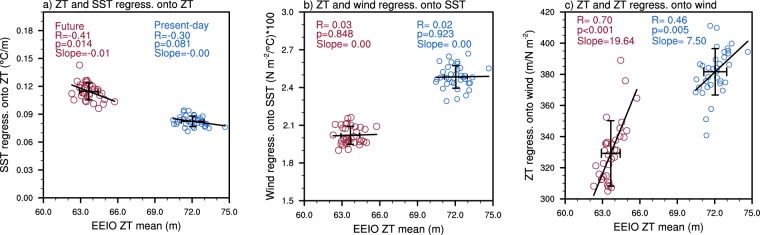
Relationship between thermocline depth and components of the Bjerknes feedback. (**a**) Present-day (blue circles) and future (red circles) SON relationship between mean EEIO thermocline depth (ZT) and the SST response to thermocline depth. (**b**,**c**) as in (**a**) but for the zonal wind stress response to SST and the thermocline response to zonal wind stress, respectively. SST and ZT are averaged over the EEIO region. The zonal wind stress has been averaged over the central equatorial Indian Ocean (CEIO). The diagonal black lines represent the line of best fit and the error bars indicate 1 standard deviation from the multi-member ensemble mean.

It is important to understand how the mean state of the thermocline influences the components of the positive Bjerknes feedback due to the vital role of the feedback in IOD evolution and growth^22,32–34^. As mentioned previously, this feedback involves the SSTA response to thermocline depth anomalies, the anomalous wind response to SSTAs, and the thermocline response to wind anomalies. The present-day SSTA response to thermocline depth anomalies (i.e., the thermocline feedback) shows a weak negative correlation with the EEIO mean thermocline depth that is significant at the 90% confidence level (Fig. 5a). This suggests that simulations with a deeper present-day mean thermocline tend to have a weaker thermocline feedback. In the future period, this relationship strengthens due to the shoaling of the mean thermocline in response to a warmer climate, which increases the sensitivity of SSTs to thermocline depth anomalies^17,18^. The anomalous wind response to SSTAs shows no relationship with mean thermocline depth in both the present-day and future periods, suggesting that this component of the Bjerknes feedback is not influenced by the mean thermocline depth (Fig. 5b). However there is a significant positive relationship (99% confidence level) between the thermocline response to zonal wind anomalies and the mean thermocline depth (Fig. 5c). These positive correlations imply that when the mean thermocline depth is deeper, the anomalous thermocline response to wind anomalies tends to be stronger. For the anomalous wind response to SSTAs, and the anomalous thermocline response to wind anomalies, the multi-member ensemble means show a weakening in the future. In a warmer climate, the stratification of the lower atmosphere increases^17,18^, leading to reduced zonal wind variance and thus a weakening of these two feedback components.

## Discussion and Summary

The influence of the mean thermocline state on IOD properties is clearly evident in the strong correlations exhibited in CESM-LE but it is important to remember that there may be biases in the model. Compared to observations, the climatological thermocline slope in CESM-LE is opposite, with the EEIO thermocline depth being considerably shallower than the WEIO region (Table S1) whereas in reality, the WEIO is slightly shallower than the EEIO^32^. This bias is not unique to CESM-LE and is common amongst CMIP3 and CMIP5 models^32^. As a result, the mean easterly winds and the zonal SST gradient are overly strong in CESM-LE and thus the Bjerknes feedback is also too strong. This causes the simulated IOD amplitude to be considerably larger than observations. Nevertheless, this bias is consistent amongst all thirty-five runs examined as the parameterisations and physics in each simulation are identical.

As each ensemble member differs only in its initial condition, where a small round-off error is applied, any difference between ensemble members arises from internal climate variability. Over the EEIO, this emerges as small differences in climatological thermocline depth and in the present-day period the difference in depth between the shallowest and deepest simulations is approximately four metres (Table S1). Although the spread amongst the thirty-five simulations is relatively small, the variations in mean thermocline depth are enough to generate significant correlations with IOD amplitude, skewness, and the mean zonal SST gradient. This highlights the importance of the mean state and the Bjerknes feedback for the IOD.

These relationships are important for projections of IOD properties and in CESM-LE the present-day climatological thermocline depth is significantly correlated with changes in IOD amplitude and skewness. It has been suggested that there is no relationship between the change in IOD amplitude and the change in the mean state of the tropical IO and this is true when examining the whole tropical basin^30^. However, in observations the IOD is dominated by the EEIO region where SST variance is largest^14,35^ and CESM-LE also exhibits this behaviour. Over the EEIO, the climatological thermocline shoals considerably more than the WEIO with the multi-member ensemble median change of −8.3 metres in the east compared to a median deepening of 0.8 metres in the west (Table S1). Therefore, by focusing on the EEIO, we are able to show that internal variability of the mean EEIO state and its response to a warmer climate can influence IOD properties.

## Methods

### Model data

The Community Earth System Model Large Ensemble (CESM-LE) is an ensemble of simulations run using a single coupled general circulation model (CGCM), the Community Earth System Model (CESM)^29^. Small differences in round-off level in the atmospheric initial conditions lead to each ensemble member having its own unique climate trajectory and therefore, the ensemble spread in CESM-LE arises from internal climate variability. CESM involves several component models; the Community Atmosphere Model version 5 (CAM5), the Parallel Ocean Program version 2 (POP2), the Community Land Model version 4 (CLM4), and the Los Alamos Sea Ice Model (CICE). These models are coupled using the version 7 coupler (CPL7). The first member of CESM-LE begins at 1850 using initial conditions from a randomly selected date in the 1850 control run. Subsequent ensemble members begin from 1 January 1920 of ensemble member 1, with member 2 using lagged ocean temperatures. Members 3–35 use slightly different initial conditions caused by small round-off differences in air temperature. The memory of this small perturbation is typically lost within weeks in the atmosphere, after which each member evolves chaotically and through random, stochastic processes^29^. All members of CESM-LE have the same external forcings which follow the CMIP5 design protocol^36^, historical forcings are applied from 1920 to 2005 and representative concentration pathway 8.5 (RCP8.5) forcing occurs from 2006 to 2100. Further information about CESM-LE and its experiment design can be found in Kay *et al*.^29^.

For this analysis, ocean and atmosphere variables from thirty-five simulations are utilised. These variables include; SST, thermocline depth (defined as the depth of the maximum vertical gradient), zonal wind stress, precipitation, and mean sea level pressure. Present-day (Future) data are restricted to the 1920 to 1999 (2020 to 2099) period and all variables have been regridded to a 1° by 1° grid. Using a different window size (e.g., 1920 to 1969 and 2050 to 2099) or shifting the window location (e.g., using periods 1930 to 1979 and 2030 to 2079) does not significantly alter the results of this analysis. The IOD is defined by the Dipole Mode Index^1^, described as area averaged SSTAs in the WEIO (10°S-10°N, 50°E-70°E) minus the EEIO (10°S-Eq., 90°E-110°E).

### Statistical analysis and significance

Linear correlation and regression are used to identify relationships between IOD properties and the mean state of the IO. To examine whether the difference between two smaller ensembles is significant, a two-tailed Student’s *t*-test is used and values greater than the 99% confidence level are considered to be significant.

### Seasonality

All calculations in this paper are performed over the austral spring season (September-November, SON), which is when the IOD peaks. CMIP5 models and CESM-LE are able to simulate the seasonal phase locking of the IOD well^20,30^.

### Graphics software

All maps and plots were produced using NCAR Command Language (NCL) version 6.4.0. 10.5065/D6WD3XH5.

## Electronic supplementary material

Supplementary Information

## Data Availability

The original CESM-LE data analysed in this study are available from the Earth System Grid repository, http://www.cesm.ucar.edu/projects/community-projects/LENS/data-sets.html. The data generated during this study are available from the corresponding author on reasonable request.
