# Supplementary material for: Influence of internal climate variability on Indian Ocean Dipole properties
Source: Sci Rep. 2018 Sep 10;8:13500. doi: 10.1038/s41598-018-31842-3 (PMC6131175; doi:10.1038/s41598-018-31842-3)
Supplement: Supplementary file 1 — Supplementary Information [file 41598_2018_31842_MOESM1_ESM.pdf]

Influence of internal climate variability on Indian Ocean Dipole properties

Benjamin Ng*[1, 2], Wenju Cai[1, 2], Tim Cowan[3], and Daohua Bi[1, 2]

[1]CSIRO Climate Science Centre

[2]Centre for Southern Hemisphere Ocean Research

[3]School of Geosciences, The University of Edinburgh

*Correspondence and requests for materials should be addressed to B. N.

(Benjamin.Ng@csiro.au)

Supplementary tables and figures

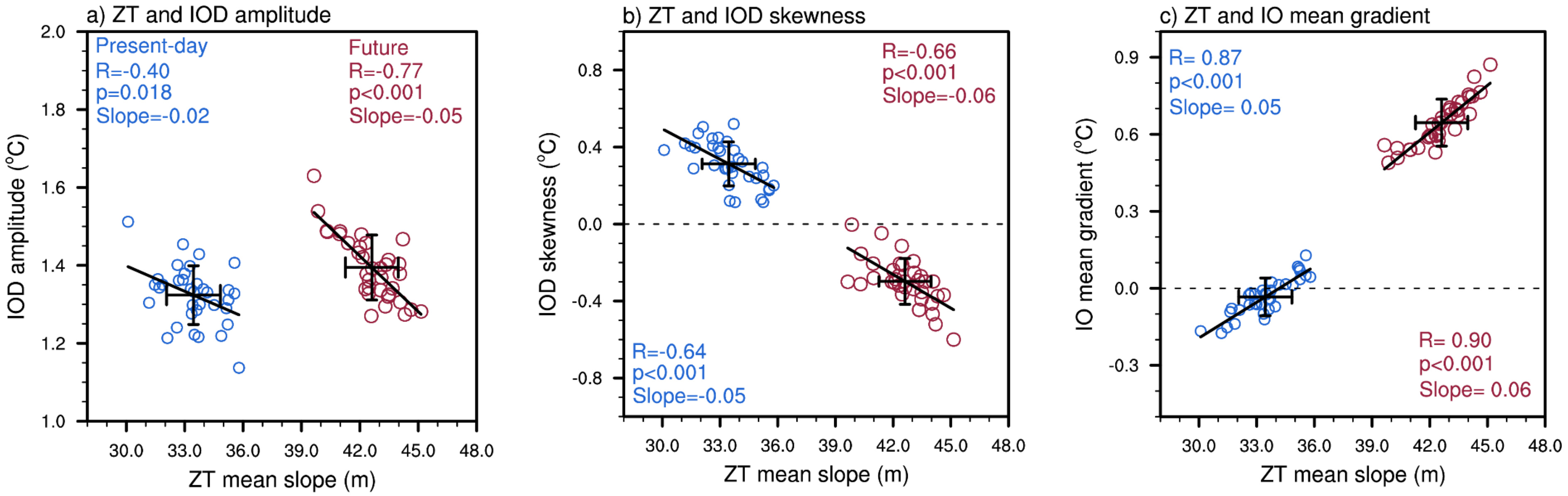

Figure S1 | Relationship between thermocline mean slope and IOD properties. (a) Present-day (blue circles) and future (red circles) SON relationship between the climatological thermocline (ZT) slope and IOD amplitude. (b), (c) As in (a) but for IOD skewness and the IO mean gradient, respectively. The slope is defined as the WEIO minus EEIO difference in thermocline depth. The diagonal black line represents the line of best fit and the error bars indicate 1 standard deviation from the multi-member ensemble mean.

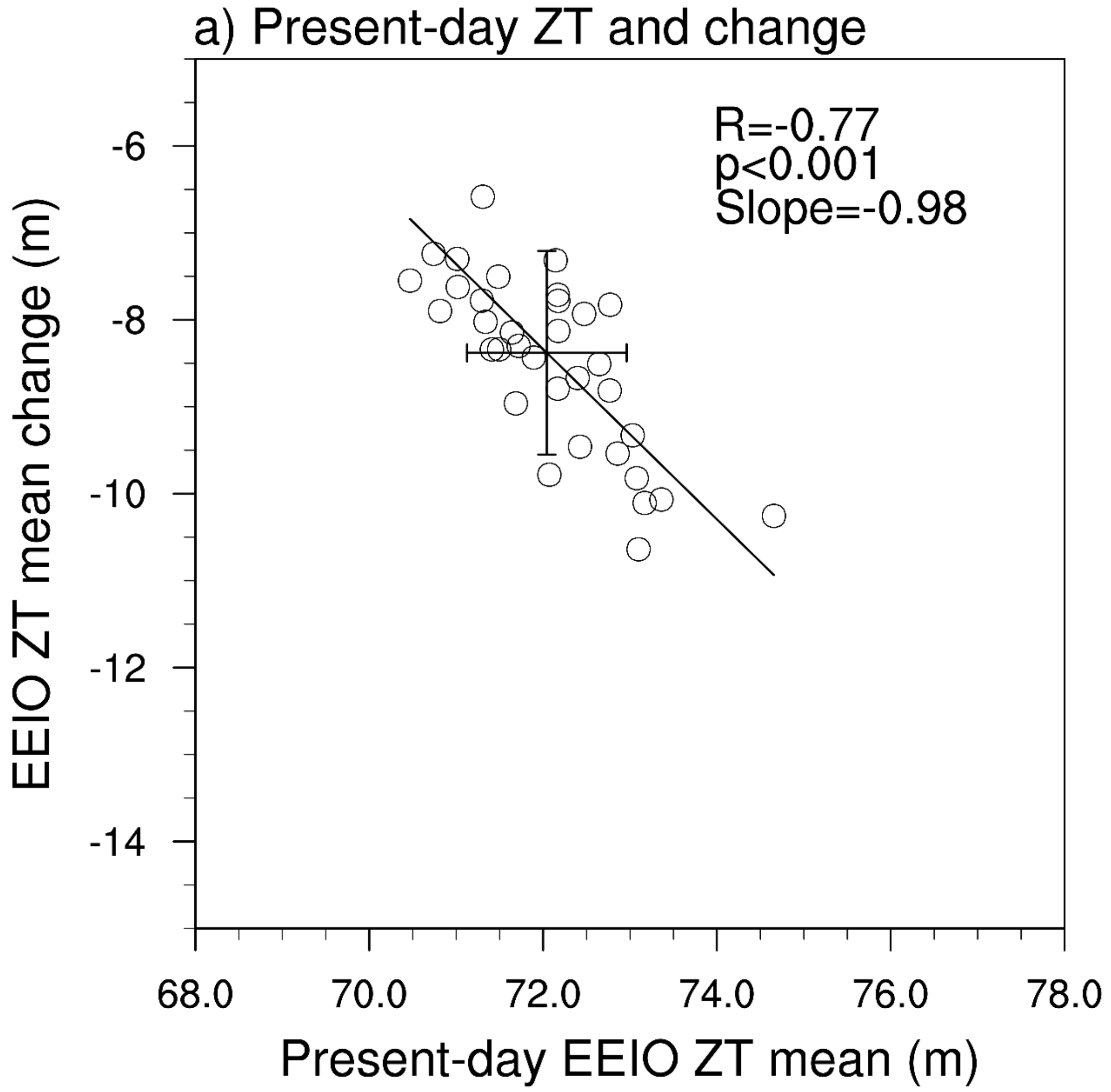

Figure S2 | Relationship between present-day EEIO mean thermocline depth and the EEIO change in thermocline depth. The diagonal black line represents the line of best fit and the error bars indicate 1 standard deviation from the multi-member ensemble mean.

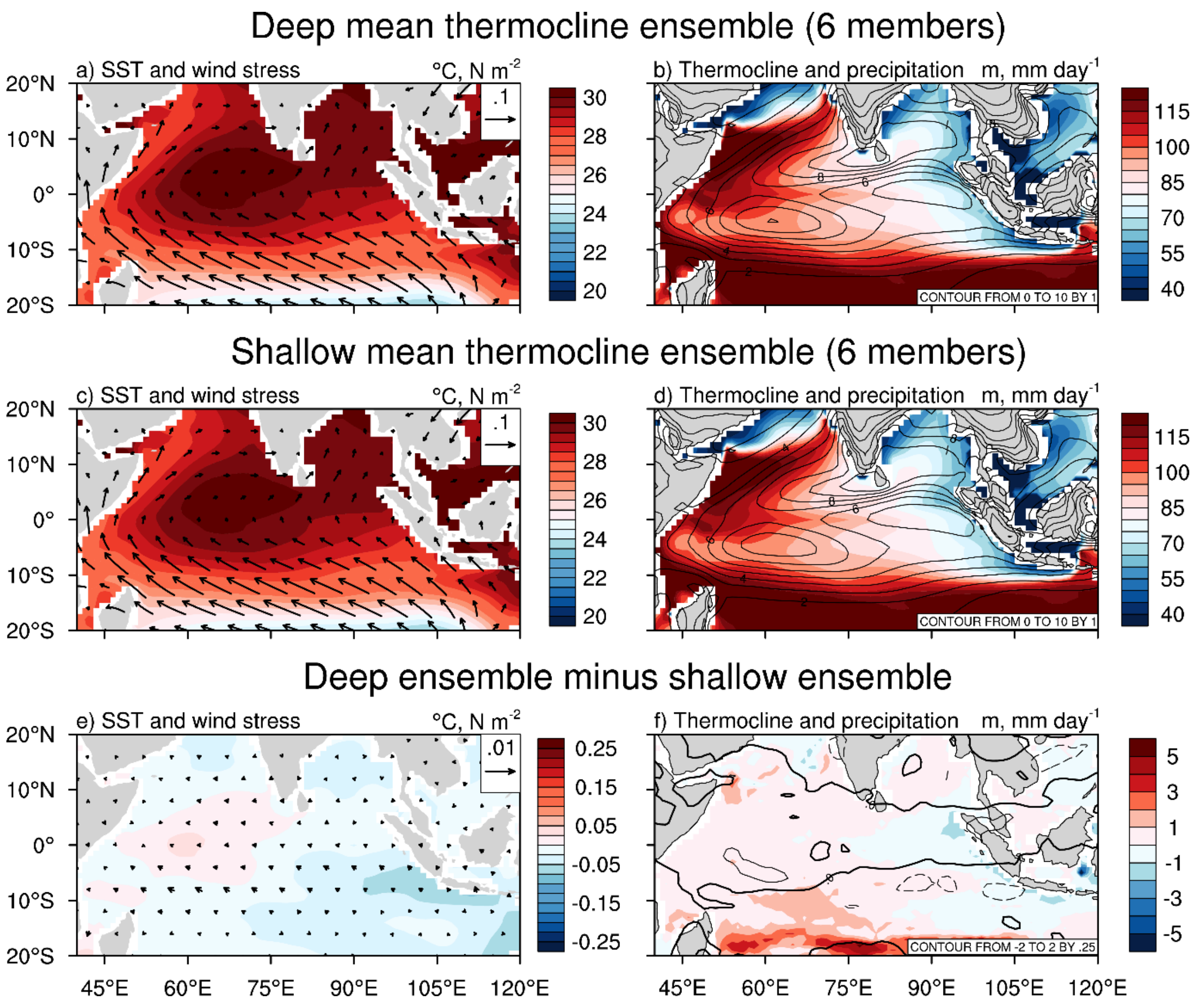

Figure S3 | Future mean state over the tropical Indian Ocean for simulations with a present-day deep or shallow thermocline. (a) Ensemble mean future climatological SSTs and wind stress from six simulations with a deep present-day mean EEIO thermocline (i.e., the same simulations used in **Figure 3**). (b) As in (a) but for thermocline depth and precipitation. (c), (d), As in (a), (b), respectively, but for six simulations with a shallow present-day mean EEIO thermocline. (e) The difference in mean SSTs and wind stress between the two six-member ensembles (i.e., (a) minus (c)). (f), As in (e) but for thermocline depth and precipitation.

Run	EEIO present-day ZT	EEIO future ZT	Present-day minus future	WEIO present-day ZT	WEIO future ZT	Present-day minus future
1	72.40	63.73	-8.67	105.10	106.36	1.26
2	71.49	63.15	-8.34	106.01	106.83	0.82
3	70.54	**65.75**	-4.79	106.33	105.39	-0.94
4	73.36	63.29	-10.07	104.53	106.40	1.87
5	72.17	64.46	-7.71	105.58	105.44	-0.14
6	72.17	64.05	-8.13	104.77	106.34	1.56
7	72.42	62.97	-9.46	105.73	106.03	0.29
8	**70.47**	62.92	-7.55	105.35	106.37	1.02
9	73.03	63.70	-9.33	104.65	106.18	1.52
10	72.17	63.38	-8.79	105.63	105.96	0.33
11	73.08	63.26	-9.82	104.54	107.27	2.73
12	70.74	63.50	-7.24	105.99	106.56	0.57
13	71.64	63.50	-8.15	105.15	105.95	0.80
14	**74.66**	64.40	-10.26	104.74	106.48	1.73
15	72.65	64.14	-8.51	105.56	106.14	0.59
16	70.81	62.92	-7.90	105.95	106.41	0.45
17	72.77	64.94	-7.83	105.40	**104.80**	-0.60
18	71.89	63.46	-8.43	105.25	105.92	0.67
19	72.18	64.39	-7.78	106.14	105.35	-0.78
20	71.41	63.07	-8.34	105.19	107.38	2.20
21	72.77	63.95	-8.81	105.77	105.89	0.12
22	71.02	63.40	-7.62	106.58	106.87	0.29
23	71.01	63.71	-7.30	106.57	106.03	-0.54
24	72.14	64.83	-7.32	105.89	105.14	-0.75
25	72.86	63.32	-9.54	105.77	106.67	0.90
26	71.34	63.31	-8.02	105.50	107.52	2.02
27	73.17	63.06	-10.11	105.04	107.12	2.08
28	71.69	62.73	-8.96	105.30	106.80	1.50
29	71.30	63.52	-7.78	106.51	105.87	-0.64
30	72.07	**62.29**	-9.78	**104.18**	106.92	2.74
31	71.48	63.98	-7.50	**106.74**	105.38	-1.36
32	71.31	64.72	-6.58	105.02	105.05	0.03
33	71.72	63.42	-8.30	105.35	106.73	1.37
34	73.10	62.46	-10.64	104.80	**107.61**	2.81
35	72.47	64.54	-7.93	105.74	106.68	0.94
**Avg.**	**72.04**	**63.66**	**-8.38**	**105.50**	**106.28**	**0.79**
**Med.**	**72.14**	**63.50**	**-8.30**	**105.50**	**106.36**	**0.80**
**Var.**	**0.84**	**0.56**		**0.41**	**0.51**	

Table S1 | SON average thermocline depth values over the eastern equatorial Indian Ocean (EEIO) and the western equatorial Indian Ocean (WEIO) for the present-day and future periods. The multi-member ensemble average, median, and variance are shown in the

last three rows. The minimum (maximum) values of thermocline depth have been highlighted in **blue** (**red**).
